# Longitudinal clinical outcomes in a real-world population of patients with idiopathic pulmonary fibrosis: the PROOF registry

**DOI:** 10.1186/s12931-019-1182-z

**Published:** 2019-10-24

**Authors:** Wim A. Wuyts, Caroline Dahlqvist, Hans Slabbynck, Marc Schlesser, Natacha Gusbin, Christophe Compere, Sofie Maddens, Yuan-Chi Lee, Klaus-Uwe Kirchgaessler, Karen Bartley, Benjamin Bondue

**Affiliations:** 1 Department of Respiratory Medicine, Unit for Interstitial Lung Diseases, University Hospitals Leuven, Leuven, Belgium; 2 CHU UCL Namur Site Godinne, Yvoir, Belgium; 3 Department of Respiratory Medicine, ZNA Middelheim, Antwerp, Belgium; 4 Department of Respiratory Medicine, Centre Hospitalier de Luxembourg, Luxembourg City, Luxembourg; 5 CHR de la Citadelle, Liège, Belgium; 6 CHIREC Hospital, Brussels, Belgium; 7 AZ Groeninge, Kortrijk, Belgium; 8 Genentech, Inc., South San Francisco, CA USA; 9 F. Hoffmann-La Roche, Ltd., Basel, Switzerland; 10 Department of Respiratory Medicine, Erasme University Hospital, Brussels, Belgium

## Abstract

**Background:**

The PROOF registry is an observational study initiated in October 2013 with the aim to monitor disease progression in a real-world population of patients with idiopathic pulmonary fibrosis (IPF). Here, we present longitudinal clinical outcomes from the PROOF registry.

**Methods:**

Patients with IPF were enrolled across eight centers in Belgium and Luxembourg. For all patients, clinical outcomes data were collected, including mortality, lung transplant, acute exacerbations, and pulmonary hypertension. For patients treated with pirfenidone at any time during follow-up (2013–2017), for any duration of treatment (the pirfenidone-treated population): pirfenidone treatment patterns were collected; changes in pulmonary function (forced vital capacity [FVC] and carbon monoxide diffusing capacity [DLco]) were reviewed up to 24 months post-inclusion; and time-to-event analyses from the time of registry inclusion were performed.

**Results:**

The PROOF registry enrolled a total of 277 patients. During follow-up, 23.1% of patients died, 5.1% received a lung transplant, 5.4% experienced an acute exacerbation, and 6.1% had comorbid pulmonary hypertension. In the pirfenidone-treated population (*N* = 233, 84.1%), 12.9% of patients had a temporary dose discontinuation and 31.8% had a temporary dose reduction; 4.3% of patients permanently discontinued pirfenidone due to an adverse drug reaction. Mean percent predicted FVC was 81.2% (standard deviation [SD] 19.0) at Month 0 and 78.3% (SD 25.0) at Month 24, and mean percent predicted DLco was 47.0% (SD 13.2) and 45.0% (SD 16.5), respectively. Rates of ≥ 10% absolute decline in percent predicted FVC and ≥ 15% absolute decline in percent predicted DLco over 24 months were 31.0% and 23.2%, respectively. Mean times from registry inclusion to categorical absolute decline in percent predicted FVC and percent predicted DLco were 20.1 (standard error [SE] 0.6) months and 23.4 (SE 0.5) months, respectively; mean time from registry inclusion to death was 31.0 (SE 0.9) months.

**Conclusions:**

The PROOF registry is a source of European data characterizing longitudinal clinical outcomes of patients with IPF. Over 12 months of follow-up, pulmonary function remained largely stable in patients with IPF who received pirfenidone for any duration of treatment. Pulmonary function remained similar at 24 months of follow-up, although patient numbers were lower.

**Trial registration:**

PROOF is registered with the relevant authorities in Belgium and Luxembourg, with registration to Comité National d’Éthique et de Recherche (CNER) N201309/03–12 September 2013 and a notification to Comité National de Protection des Données (CNDP) for Luxembourg.

## Background

Idiopathic pulmonary fibrosis (IPF) is a progressive, irreversible, debilitating, fibrosing lung disease that is associated with survival rates lower than those reported for many cancers [1–3]. Patients with IPF experience progressive worsening of pulmonary function and reductions in exercise capacity, with a variable rate of decline between patients [2, 3].

Two antifibrotics, pirfenidone and nintedanib, which were approved for the treatment of patients with IPF in Europe in 2011 and 2015, respectively, have been shown to reduce the rate of disease progression in patients with IPF [4–7]. In a pooled analysis of three phase III clinical trials, ASCEND (Study 016; NCT01366209) and CAPACITY (Studies 004 and 006; NCT00287716 and NCT00287729), pirfenidone was shown to significantly reduce the decline in lung function and exercise capacity observed over 52 weeks compared with placebo [6]. In the INPULSIS trials (INPULSIS 1 and 2; NCT01335464 and NCT01335477), nintedanib was shown to significantly reduce lung function decline over 52 weeks compared with placebo [7].

Although these trials provide important information regarding the efficacy and safety of antifibrotics in patients with IPF, the strict inclusion and exclusion criteria may limit generalization of the results to real-world populations of patients. For example, patients with percent predicted forced vital capacity (FVC) < 50% were excluded from ASCEND, CAPACITY, and INPULSIS, as were patients with percent predicted carbon monoxide diffusing capacity (DLco) < 30% (ASCEND and INPULSIS) or < 35% (CAPACITY) [7–9]. In addition, patients with comorbid conditions, including unstable or deteriorating cardiac or pulmonary disease, or those who were receiving certain prescribed medications were excluded from trials. In contrast, evidence from real-world populations of patients with IPF suggests that there is a high burden of comorbid conditions and concomitant medication use in clinical practice [10, 11]. Rates of treatment adherence might also differ between clinical trials and real-world populations.

Findings from real-world patient registries may be more representative of clinical practice compared with findings from clinical trials. In recent years, a number of IPF registries have been established to monitor long-term outcomes and disease progression in real-world populations of patients with IPF [12–17]. However, longitudinal clinical outcomes are only just starting to emerge [14, 18–22]. Longitudinal patient registries can play an important role in providing long-term clinical data on the course of IPF and the impact of treatment in the real-world setting [23–25]. Furthermore, longitudinal registry data can be used to investigate the relationship between changes in clinical measurements and subsequent outcomes in patients with IPF, for example, the relationship between changes in pulmonary function and exercise capacity and subsequent mortality [26–28].

The PROOF registry was initiated in October 2013 to monitor disease progression in a real-world population of patients with IPF [29, 30]. Here, we present longitudinal findings from the PROOF registry from October 2013 to July 2017, including clinical outcomes in all patients with IPF, and changes in pulmonary function in patients with IPF treated with pirfenidone.

## Methods

### Registry design

The PROOF registry is an observational study which aims to monitor disease progression in a real-world population of patients with IPF.

Patients were enrolled across seven centers in Belgium and one center in Luxembourg during the period of October 2013 to January 2016. The majority of IPF diagnoses took place at centers with a highly experienced multidisciplinary team (MDT) present. Patients eligible for inclusion in the PROOF registry were over 18 years of age and had an MDT diagnosis of definite or probable IPF according to 2011 American Thoracic Society/European Respiratory Society/Japanese Respiratory Society/Latin American Thoracic Association guidelines [3]. Patients with a history of environmental exposures could be eligible for inclusion if MDT discussions excluded hypersensitivity pneumonitis or other interstitial lung diseases with known cause. Patients were excluded if they were enrolled in a clinical trial at the time of inclusion in the PROOF registry.

The PROOF registry was conducted in accordance with the International Council on Harmonisation of Technical Requirements for Registration of Pharmaceuticals for Human Use, Guidelines for Good Clinical Practice, and local legal and regulatory requirements. Patients were required to provide informed consent prior to inclusion.

### Analysis populations

Patients were enrolled between October 2013 and January 2016. Analyses were performed at a cut-off date of July 2017 and included all patients with 24 months of data available. For assessment of clinical outcomes, the analysis population included all patients with IPF included in the PROOF registry. For pirfenidone treatment patterns, longitudinal changes in pulmonary function, and time-to-event analyses, the pirfenidone-treated population was used, which consisted of patients treated with pirfenidone at any time during follow-up in the registry (at the time of registry inclusion or after registry inclusion), for any duration of treatment. Longitudinal data for patients treated with nintedanib were not assessed due to low patient numbers (enrollment into the PROOF registry began prior to the introduction of nintedanib).

### Patient demographics and clinical outcomes

Demographic data, including gender, race, age, smoking status, previous treatment for IPF other than pirfenidone, and supplemental oxygen use, were collected upon inclusion in the PROOF registry for all patients. Comorbidities and co-medications recorded at registry inclusion, such as emphysema and antihypertensives, are reported in detail in the PROOF baseline manuscript [30].

Clinical outcomes reported at any point during follow-up were collected for all patients. These outcomes included mortality, lung transplant, acute exacerbation (defined as an acute, clinically significant deterioration of unidentifiable cause in a patient with underlying IPF) [31], and comorbid pulmonary hypertension (defined at the clinician’s discretion and in most cases based on systolic pulmonary arterial pressure [≥ 35 mmHg] on echocardiography. Right heart catheterization was performed in a minority of patients).

### Pirfenidone treatment patterns and longitudinal changes in pulmonary function

Pirfenidone treatment patterns were collected; these patterns included permanent and temporary discontinuations and temporary dose reductions, each with reasons. Times to temporary discontinuation and temporary dose reduction were also reviewed, but due to the high proportion of patients with incomplete data (since they initiated pirfenidone prior to entering the registry), these data are not reported.

For the pirfenidone-treated population, longitudinal changes in pulmonary function were reviewed up to 24 months post-inclusion. Pulmonary function measures included percent predicted FVC and percent predicted DLco. FVC and DLco were assessed at inclusion (Month 0) and at Months 3, 6, 12, and 24. The percentages of patients experiencing a ≥ 10% decline in percent predicted FVC compared with inclusion (Month 0) were calculated at Months 3, 6, 12, and 24. Similarly, the percentages of patients experiencing a ≥ 15% decline in percent predicted DLco were calculated at the same time points. Absolute changes in pulmonary function were the primary longitudinal evaluation, with relative changes also determined. Longitudinal changes in exercise capacity, assessed using the 6-min walk test, were also reviewed up to 24 months post-inclusion, but the high proportion of patients with missing values preclude reporting of the data.

### Data analysis

A programmed database received all information collected in the electronic case report forms, and automated edit checks were conducted. A contract research organization was responsible for the management of data, including data quality checks, and was also required to produce a Data Review Strategy to highlight the quality-check method performed on the data. An extensive quality control audit was conducted to ensure data quality.

For all patients, demographics and clinical outcomes were summarized descriptively.

For the pirfenidone-treated population, pirfenidone treatment patterns were summarized descriptively. Longitudinal pulmonary function was also summarized descriptively, with patients included in the calculation if they had a lung function measurement available for the specified time point, regardless of whether they had a baseline measurement. For mean changes in pulmonary function from Month 0 and the percentages of patients experiencing a categorical decline in FVC or DLco from Month 0, patients were included in the calculation for a time point if they had an FVC or DLco measurement available for that time point and a corresponding measurement at Month 0. For each analysis, patients with missing data for the required time points were excluded. Kaplan–Meier time-to-event curves were constructed to summarize estimates for ≥ 10% absolute decline in percent predicted FVC, ≥ 15% absolute decline in percent predicted DLco, and death, each from the time of registry inclusion, and also to investigate these outcomes in patients who experienced a temporary dose reduction or discontinuation of pirfenidone and in patients who did not.

Slope analysis was conducted to estimate intra-individual annual declines in percent predicted FVC before and after pirfenidone treatment. This analysis extended past 24 months because some patients had data over an observation period of more than 24 months at the time of cut-off, depending on their date of enrollment. All available data were used in the slope analysis and there was no imputation for missing values. The slope for annual rate of FVC decline was analyzed using a random coefficient regression (random slopes and intercepts) model, with country as covariate. Percent predicted FVC data available from 3 years before to 5 years after the first pirfenidone treatment were included in the slope analysis. A sensitivity analysis was performed including only patients with a known date of pirfenidone initiation, i.e. patients who initiated pirfenidone at or after inclusion in the PROOF registry.

## Results

### Patient demographics

The PROOF registry enrolled 277 patients with IPF between October 2013 and January 2016. Eight patients were classified as incident cases, defined as a date of diagnosis on or after the date of inclusion in the registry. Although MDT diagnosis was an inclusion criterion for the registry, six patients had not undergone MDT diagnosis and data were missing for one patient. A total of 233 patients were treated with pirfenidone at any time during follow-up, for any duration of treatment. Of these 233 patients, 162 had already initiated treatment with pirfenidone at the time of registry inclusion and 71 patients initiated treatment with pirfenidone after registry inclusion. The remaining 44 patients enrolled in the PROOF registry did not receive pirfenidone at any time during follow-up. The mean time between diagnosis and initiation of pirfenidone treatment was 536.8 (standard deviation [SD] 745.2) days. A total of 28 patients were treated with nintedanib at any time during follow-up in the PROOF registry; of these, 26 had previously received pirfenidone.

The majority of the 277 patients with IPF included in the registry were male (76.9%), white (92.1%), and a former/current (66.8%/6.5%) smoker (Table 1). Mean age was 69.6 (SD 8.6) years. Previous treatment for IPF other than pirfenidone is shown in Table 1; nine patients in total had previously received nintedanib (four patients in the pirfenidone-treated population and five patients who had never received pirfenidone). Further details on demographic and baseline characteristics of this patient population have been recently reported [30].

**Table 1:** Patient demographics at registry inclusion

Parameter^a^	All patients	Patients ever treated with pirfenidone^b^	
	(*N* = 277)	Yes (*N* = 233)	No (*N* = 44)
Male	213 (76.9)	181 (77.7)	32 (72.7)
White	255 (92.1)	219 (94.0)	36 (81.8)
Age, years	Mean 69.6 (SD 8.6)	Mean 69.4 (SD 8.5)	Mean 70.6 (SD 8.9)
Smoking status			
Never	74 (26.7)	65 (27.9)	9 (20.5)
Current	18 (6.5)	15 (6.4)	3 (6.8)
Former	185 (66.8)	153 (65.7)	32 (72.7)
Previous treatment for IPF other than pirfenidone			
Nintedanib	9 (3.3)	4 (1.7)	5 (11.4)
N-acetylcysteine	68 (24.6)	62 (26.6)	6 (13.6)
Corticosteroids	50 (18.1)	46 (19.7)	4 (9.1)
Azathioprine	3 (1.1)	2 (0.9)	1 (2.3)
Ambrisentan	1 (0.4)	0 (0.0)	1 (2.3)
Supplemental oxygen use	29 (10.5)	25 (10.7)	4 (9.1)

^a^Data are presented as *n* (%) unless otherwise specified

^b^Patients treated with pirfenidone at any time during follow-up in the registry (at the time of registry inclusion or after registry inclusion), for any duration of treatment

*IPF* idiopathic pulmonary fibrosis, *SD* standard deviation

### Clinical outcomes

Of the 277 patients included in the registry, 23.1% (*n* = 64) died during the follow-up period, with 70.3% (45/64) of the deaths considered to be related to IPF. Acute exacerbations (as decided on a clinical basis) were reported for 5.4% (*n* = 15) of patients and comorbid pulmonary hypertension for 6.1% (*n* = 17) of patients. Lung transplant was reported for 5.1% (*n* = 14) of patients.

### Pirfenidone treatment patterns

Of the 233 patients in the pirfenidone-treated population, 63.1% (*n* = 147) left the registry and rolled over to the extension study registry (Table 2). Reasons for permanent pirfenidone discontinuation in the registry during follow-up included death in 22.7% (*n* = 53) of patients, lung transplant in 5.6% (*n* = 13) of patients, and an adverse drug reaction (ADR) in 4.3% (*n* = 10) of patients. The remaining permanent pirfenidone discontinuations (4.3% [*n* = 10] of patients) were for another or unknown reason.

**Table 2:** Pirfenidone treatment patterns during follow-up

Parameter^a^	Pirfenidone-treated population
	(*N* = 233)
Rollover to an extension study	147/233 (63.1)
Patients who experienced permanent pirfenidone discontinuation	86/233 (36.9)
Death	53/233 (22.7)
Lung transplant	13/233 (5.6)
ADR	10/233 (4.3)
Lost to follow-up	5/233 (2.2)
Other/unknown	5/233 (2.2)
Patients who experienced temporary pirfenidone discontinuation	30/233 (12.9)
Any ADR	8/30 (26.7)
Gastrointestinal ADR	4/30 (13.3)
Skin ADR	3/30 (10.0)
Fatigue	1/30 (3.3)
Other/unknown	22/30 (73.3)
Patients who experienced temporary pirfenidone dose reduction	74/233 (31.8)
ADR	21/74 (28.4)
Gastrointestinal ADR	12/74 (16.2)
Skin ADR	6/74 (8.1)
Fatigue	3/74 (4.1)
Other ADR	1/74 (1.4)
Other/unknown	53/74 (71.6)

^a^Data are presented as *n*/*N* (%)

*ADR* adverse drug reaction

Pirfenidone treatment was temporarily discontinued in 12.9% (30/233) of patients (Table 2). Around one-quarter of the temporary discontinuations (26.7%; 8/30) were reported to be due to an ADR, including a gastrointestinal ADR in four patients (13.3%; 4/30), a skin ADR in three patients (10.0%; 3/30), and fatigue in one patient (3.3%; 1/30). The remaining three-quarters of temporary discontinuations (73.3%; 22/30 cases) were reported to be for a non–ADR-related or unknown reason.

Temporary pirfenidone dose reductions occurred in 31.8% (74/233) of patients (Table 2). ADRs were reported to be the reason for dose reduction in 28.4% (21/74) of cases, with gastrointestinal ADRs, skin ADRs, and fatigue responsible for dose reduction in 12, 6, and 3 patients, respectively. The remaining 71.6% of dose reductions (53/74) were reported to be for a non–ADR-related or unknown reason.

### Longitudinal changes in pulmonary function in pirfenidone-treated patients

**Fig. 1:**
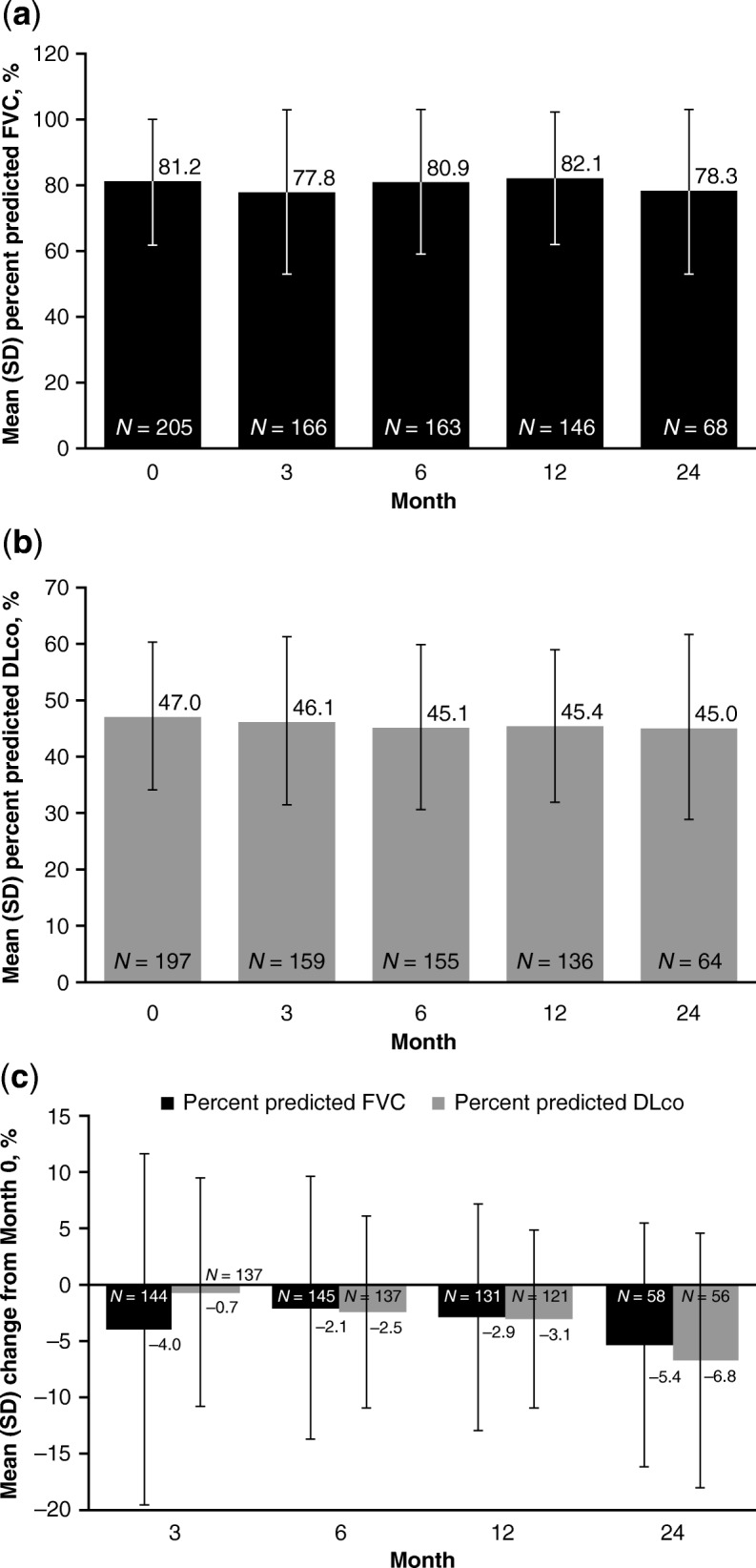
Pulmonary function over 24 months (pirfenidone-treated population) (**a**) Mean (SD) percent predicted FVC, (**b**) Mean (SD) percent predicted DLco, and (**c**) Mean (SD) change from Month 0. Month 0 is the time of inclusion in the PROOF registry. Patients were included in the calculation if they had a lung function measurement available for the specified time point, regardless of whether they had a baseline measurement (**a**, **b**). For mean changes in pulmonary function from Month 0, patients were included in the calculation for a time point if they had an FVC or DLco measurement available for that time point and a corresponding measurement at Month 0 (**c**). *DLco* carbon monoxide diffusing capacity, *FVC* forced vital capacity, *SD* standard deviation

In the pirfenidone-treated population, at Month 0, mean percent predicted FVC was 81.2% (SD 19.0; *n* = 205) (Fig. 1a) and mean percent predicted DLco was 47.0% (SD 13.2; *n* = 197) (Fig. 1b). At Month 24, mean percent predicted FVC was 78.3% (SD 25.0; *n* = 68) (Fig. 1a) and 31.0% (18/58) of patients had experienced a ≥ 10% absolute decline in percent predicted FVC compared with Month 0 (Table 3). Mean percent predicted DLco at Month 24 was 45.0% (SD 16.5; *n* = 64) (Fig. 1b) and 23.2% (13/56) of patients had experienced a ≥ 15% absolute decline in percent predicted DLco compared with Month 0 (Table 3). When mean percent predicted FVC at each time point was calculated separately for patients who were alive at Month 24 and patients who had died by Month 24, lung function was largely stable in both groups over time, but patients who survived showed higher mean percent predicted FVC at each time point (Additional file 1: Figure S1). Mean absolute changes from Month 0 in percent predicted FVC and percent predicted DLco are shown in Fig. 1c.

**Table 3:** Percentage of patients experiencing categorical absolute decline over 24 months from Month 0 (pirfenidone-treated population)

	Month 3	Month 6	Month 12	Month 24
Absolute FVC decline ≥ 10%, *n*/*N* (%)	16/144 (11.1)	24/145 (16.6)	25/131 (19.1)	18/58 (31.0)
Absolute DLco decline ≥ 15%, *n*/*N* (%)	6/137 (4.4)	9/137 (6.6)	8/121 (6.6)	13/56 (23.2)

*DLco* carbon monoxide diffusing capacity, *FVC* forced vital capacity

Results for relative declines in percent predicted FVC and percent predicted DLco are available in Additional file 1: Table S1.

### Time-to-event analyses in pirfenidone-treated patients

**Fig. 2:**
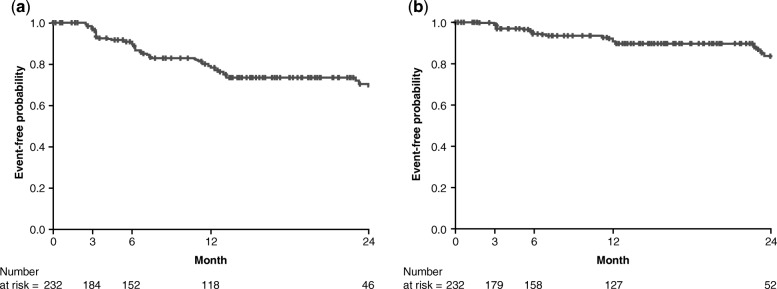
Time-to-event analyses, from registry inclusion (pirfenidone-treated population), for patients who experienced (**a**) ≥ 10% absolute decline in percent predicted FVC and (**b**) ≥ 15% absolute decline in percent predicted DLco. *DLco* carbon monoxide diffusing capacity, *FVC* forced vital capacity

Time-to-event analyses conducted in the pirfenidone-treated population for patients who experienced a ≥ 10% absolute decline in percent predicted FVC and a ≥ 15% absolute decline in percent predicted DLco are presented in Fig. 2a and b, respectively. The mean time from registry inclusion to categorical absolute decline in percent predicted FVC was 20.1 (standard error [SE] 0.6) months and the mean time from registry inclusion to categorical absolute decline in percent predicted DLco was 23.4 (SE 0.5) months. In patients with a temporary dose reduction or discontinuation of pirfenidone, mean times from registry inclusion to a ≥ 10% absolute decline in percent predicted FVC or a ≥ 15% absolute decline in percent predicted DLco were 19.2 (SE 1.1) months and 22.3 (SE 0.8) months, respectively. In patients who did not experience a temporary dose reduction or pirfenidone discontinuation, the respective values were 20.3 (SE 0.7) months and 23.5 (SE 0.5) months.

**Fig. 3:**
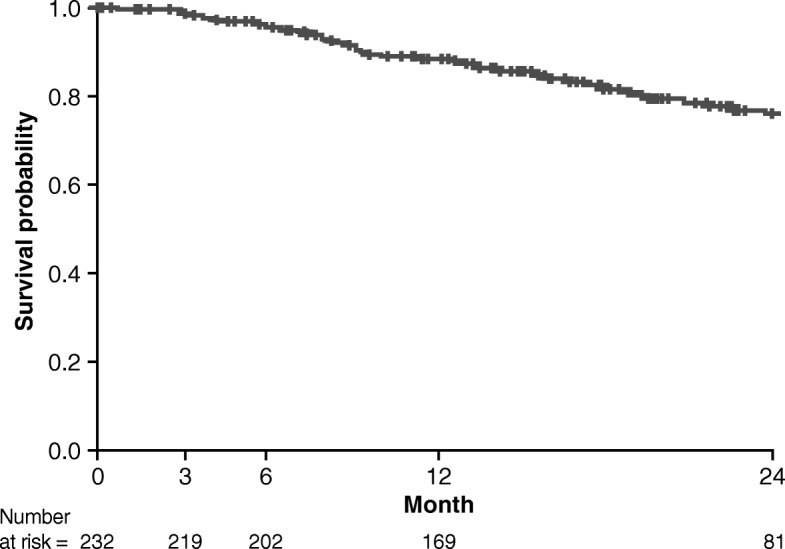
Time-to-event analysis, from registry inclusion, for patients who died (pirfenidone-treated population)

The time-to-death analysis conducted in the pirfenidone-treated population is presented in Fig. 3. The mean time from registry inclusion to death was 31.0 (SE 0.9) months. In patients with a temporary dose reduction or discontinuation of pirfenidone, the mean time from registry inclusion to death was 29.9 (SE 1.5) months. In patients without a temporary dose reduction or pirfenidone discontinuation, the corresponding value was 27.5 (SE 0.8) months. The median survival time could not be calculated because more than 50% of patients survived during the total observation period.

### Slope analysis for percent predicted FVC

**Fig. 4:**
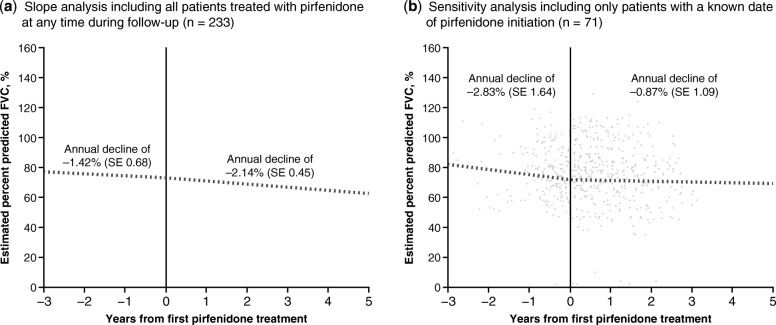
Percent predicted FVC decline 3 years before and 5 years after pirfenidone initiation (pirfenidone-treated population). (**a**) Slope analysis of the decline in percent predicted FVC in the 3 years before and 5 years after initiation of pirfenidone treatment and (**b**) Sensitivity analysis of the decline in percent predicted FVC in the population of patients who initiated pirfenidone treatment at or after inclusion in the PROOF registry. *FVC* forced vital capacity, *SE* standard error

In order to analyze the effect of treatment initiation on loss of pulmonary function and to take into consideration the intrinsic variability of lung function tests, a slope analysis of the evolution of FVC was performed, comparing the slope before and after initiation of pirfenidone (Fig. 4a; *n* = 233). The estimated percent predicted FVC at 3 years prior to first pirfenidone treatment was 77.2%. At the time of first pirfenidone treatment, the estimated percent predicted FVC was 72.9%. The estimated decline in percent predicted FVC between 3 years prior to the first treatment and the first treatment was − 1.42% (SE 0.68) per year. At 5 years after the first pirfenidone treatment, the estimated percent predicted FVC was 62.2%. The estimated decline in percent predicted FVC between the first treatment and 5 years after the first treatment was − 2.14% (SE 0.45) per year.

In a sensitivity analysis including only patients with a known date of pirfenidone initiation (Fig. 4b; *n* = 71), the estimated decline in percent predicted FVC between 3 years prior to the first treatment and the first treatment was − 2.83% (SE 1.64) per year. The estimated decline in percent predicted FVC between the first treatment and 5 years after the first treatment was − 0.87% (SE 1.09) per year.

## Discussion

The PROOF registry provides valuable evidence from a real-world population of patients with IPF in Europe. Unlike the strict inclusion and exclusion criteria employed in clinical trials, the PROOF registry did not exclude patients on the basis of disease severity, comorbidities, or prescribed medications. As a result, data from PROOF, as with other registries, can be considered to be more representative of clinical practice than data from clinical trials.

Of the total 277 patients enrolled in the PROOF registry, 233 (84.1%) were treated with pirfenidone at any time during the follow-up period (October 2013 to July 2017). A total of 28 patients were treated with nintedanib at any time during follow-up, of whom 26 had previously received pirfenidone. The high proportion of patients prescribed pirfenidone compared with nintedanib was largely driven by the fact that while pirfenidone was approved for the treatment of patients with IPF in Europe in 2011, nintedanib was not approved until 2015 [4, 5]. Enrollment into the PROOF registry began in October 2013, prior to the introduction of nintedanib and at a time when pirfenidone treatment was reimbursed in patients with ‘mild-to-moderate’ IPF in Belgium and Luxembourg. In Belgium, where the registry was primarily conducted, the prescribing of antifibrotics is limited to centers highly experienced in the clinical diagnosis and management of IPF and which have an experienced MDT available. In Luxembourg, nintedanib became available for any center to prescribe from June 2015.

Over 12 months of follow-up, in the pirfenidone-treated population, mean percent predicted FVC and DLco values remained generally stable. Pulmonary function remained similar at 24 months of follow-up, although it should be noted that patient numbers were lower. Less than one-third of patients experienced absolute declines of ≥ 10% in percent predicted FVC or ≥ 15% in percent predicted DLco at 24 months. Mean changes from Month 0 in percent predicted FVC and percent predicted DLco were < 10% at each interval of follow-up and are largely comparable with the mean changes reported in ASCEND and CAPACITY [8, 9]. The mean times to categorical absolute decline in percent predicted FVC and percent predicted DLco were around 20 months and 23 months, respectively. Mean time to a categorical decline in FVC or DLco was slightly longer in patients with uninterrupted pirfenidone treatment compared with patients who experienced a temporary dose reduction or discontinuation. It should be noted that comparisons between the pirfenidone-treated and non–pirfenidone-treated populations were not possible due to the low number of patients who had not been treated with pirfenidone (five of whom had previously been treated with nintedanib).

The results of the slope analysis were unexpected, with the rate of annual FVC decline appearing to be slightly higher after the initiation of pirfenidone versus before treatment. One potential explanation is that because many patients initiated treatment prior to enrollment (69.5% [162/233] of patients in the pirfenidone-treated population), it is possible that the dates of treatment initiation were not accurately recorded or that not all measurements of FVC were captured. To explore this hypothesis further, a sensitivity analysis was performed including only patients with a known date of pirfenidone initiation, i.e. those patients who initiated pirfenidone at or after inclusion in the PROOF registry. As expected, in the sensitivity analysis, the estimated rate of FVC decline was reduced after the initiation of pirfenidone compared with the estimated rate of FVC decline calculated for prior to pirfenidone therapy. These findings suggest that the results of the slope analysis were indeed affected by the availability and accuracy of the data recorded prior to the PROOF registry, thus supporting the body of evidence showing that pirfenidone can significantly reduce FVC decline in patients with IPF.

In addition to providing information on disease progression, the PROOF registry is a source of European data characterizing other clinically important outcomes in patients with IPF, irrespective of their treatment. Other European IPF registries with any available longitudinal clinical outcomes include the German INSIGHTS-IPF registry (*N* = 625; conducted from end-2012 to September 2015; 40.9% use of pirfenidone and 4.8% use of nintedanib by September 2015) [18] and the European IPF registry (*N* = 525; conducted November 2009 to October 2016; 95% use of antifibrotics in 2016) [14]. In the PROOF registry, among all 277 patients enrolled, the mortality rate during the follow-up period of October 2013 to July 2017 was 23.1%, with around 70% of the deaths considered to be related to IPF. Since different IPF registries have different follow-up periods, it is not possible to directly compare clinical outcome findings. However, the German INSIGHTS-IPF registry reported an annualized mortality rate of 14.2%, after a mean follow-up period of 1.2 (SD 0.7) years [18] and the European IPF registry reported a mortality rate of 38% during the follow-up period of November 2009 to October 2016 [14]. In the PROOF registry, mean survival from the time of registry inclusion in the pirfenidone-treated population was around 31 months. In the European IPF registry, median survival from the time of IPF diagnosis in the population of patients treated with an antifibrotic was around 123 months; however, this outcome is not directly comparable with the PROOF registry, as mean survival cannot be compared with median survival, and survival was calculated from registry inclusion in PROOF and from time of diagnosis in the European IPF registry [14].

In the PROOF registry, the lung transplant rate for all patients during follow-up was 5.1%. The INSIGHTS-IPF registry reported an annualized lung transplant rate of 4.9% [18], the European IPF registry reported a lung transplant rate during follow-up of 3.9% [14], and the Czech IPF registry (a national registry within an international multicenter database of patients with IPF in Central and Eastern Europe [EMPIRE]) reported a lung transplant rate during follow-up of 1.8% [22]. Acute exacerbations have been identified as an important outcome associated with an increased risk of mortality in patients with IPF [32]. In the PROOF registry, the acute exacerbation rate for all patients during follow-up was 5.4%. A total of 18.3% of patients who received pirfenidone in the Czech IPF registry were hospitalized due to an acute exacerbation; however, longitudinal data on acute exacerbations from other IPF registries are currently lacking [22]. One of the difficulties in collecting data on acute exacerbations is the lack of a standardized definition. In 2016, Collard et al. published an international working report on acute exacerbations of IPF, but prior to this, and for the majority of the PROOF registry duration, acute exacerbations were allocated using the definition in Collard et al. 2007, which did not include clear objective clinical criteria and largely left diagnosis at clinicians’ discretion [31, 33]. The PROOF registry is also currently the only IPF registry to provide longitudinal data on pulmonary hypertension, with a rate of 6.1% for all patients during follow-up. As well as being a frequent comorbidity, the presence of pulmonary hypertension may impact on the disease course and has been associated with a higher risk of mortality in patients with IPF [3]. As the symptoms of pulmonary hypertension overlap with those of IPF, many patients are not evaluated for pulmonary hypertension [34, 35]. In 2016, the European Society of Cardiology and European Respiratory Society published guidelines for the diagnosis and treatment of pulmonary hypertension, defined as mean pulmonary arterial pressure ≥ 25 mmHg at rest [36]. However, because many patients were enrolled in the PROOF registry prior to the publication of these guidelines, diagnosis of pulmonary hypertension was at the clinician’s discretion [36]. Indeed, the lack of standardized guidance, combined with the fact that many patients were not evaluated for pulmonary hypertension, may have resulted in an underestimation of the prevalence in the PROOF registry.

Among the 233 patients in the PROOF registry treated with pirfenidone at any time during follow-up, 31.8% of patients had a temporary dose reduction and 12.9% had a temporary discontinuation, with an ADR being the most frequently known reason in each case. In a pooled analysis of the phase III ASCEND and CAPACITY trials of pirfenidone in IPF, 60% of patients had a temporary dose reduction and 39% of patients had a temporary dose discontinuation [37]. The discrepancy in findings from the PROOF registry and clinical trial data may be due to incomplete data capture in PROOF, given that patients could initiate treatment prior to enrolling in the PROOF registry. In clinical practice, dose adjustments can be used to manage ADRs and support treatment persistence in patients with IPF treated with antifibrotics. For example, in a single-center, retrospective, observational study including 351 patients treated with pirfenidone, dose reduction was performed in response to 20% of adverse events [38]. Furthermore, in the real-world PASSPORT safety registry, which included 1009 patients treated with pirfenidone, dose adjustment appeared to reduce the proportion of patients discontinuing treatment, with 32% of patients with a dose adjustment subsequently discontinuing treatment due to an ADR compared with 44% of patients who did not have a dose adjustment [39]. In the PROOF registry, only 4.3% of patients treated with pirfenidone permanently discontinued treatment due to an ADR excluding deaths, although it should be noted that the cause of permanent discontinuation was not known in five patients (2.1%).

There are several potential limitations of the PROOF registry [30] that should be considered when interpreting the longitudinal outcomes presented in this analysis. With the exception of eight patients, all patients were diagnosed with IPF prior to enrollment in the registry, therefore the diagnostic data may have been affected by recall bias and it is possible that enrollment was affected by selection bias. Enrollment and survival in the registry may also have been biased by the exclusion of patients enrolled in a clinical trial, who would be expected to have less severe disease and fewer comorbidities compared with those patients who were not eligible for a clinical trial. The registry included a relatively small population of patients with IPF across a limited geographical area. Several planned analyses included patient numbers that were too low to warrant reporting (e.g. longitudinal changes in exercise capacity). Since initiation and follow-up of antifibrotic agents in Belgium are limited to centers with experience in the clinical diagnosis and management of IPF (although this is not the case in Luxembourg), results from the PROOF registry may not be representative of populations of patients with IPF in different countries. Moreover, diagnosis at a center with an experienced MDT present may not be representative of the real world. Some patients with IPF who have comorbid life-threatening conditions may not be referred to a center of excellence for the treatment of IPF due to concerns regarding treatment tolerability or a lack of licensed treatments; this may affect the comparison of results from the PROOF registry with other real-world patient populations. It is also possible that the high burden of comorbidities reported in the PROOF registry [30] may have affected patient survival. Finally, the PROOF registry was not designed as a clinical trial and so comparisons between different treatments with respect to outcomes may not be possible; in addition, the number of patients who had not been treated with pirfenidone was too low for comparison with the pirfenidone-treated population.

## Conclusion

In conclusion, the PROOF registry provides real-world data on longitudinal clinical outcomes in patients with IPF. Over 12 months of follow-up in the PROOF registry, pulmonary function remained largely stable in patients with IPF who received pirfenidone for any duration of treatment. Pulmonary function remained similar at 24 months of follow-up, although patient numbers were lower. Only 4.3% of patients permanently discontinued pirfenidone due to an ADR, a finding that supports that ADRs associated with pirfenidone can be effectively managed.

## Supplementary information

**Additional file 1: Table S1.** Percentage of patients with categorical relative decline in percent predicted FVC and DLco over 24 months compared with Month 0 (pirfenidone-treated population); **Figure S1.** Mean percent predicted FVC over time in patients that survived and in patients that had died at Month 24 (pirfenidone-treated population).

## Data Availability

Qualified researchers may request access to individual patient-level data through the clinical study data request platform (www.clinicalstudydatarequest.com). Further details on Roche’s criteria for eligible studies are available here (https://clinicalstudydatarequest.com/Study-Sponsors/Study-Sponsors-Roche.aspx). For further details on Roche’s Global Policy on the Sharing of Clinical Information and how to request access to related clinical study documents, see here (https://www.roche.com/research_and_development/who_we_are_how_we_work/clinical_trials/our_commitment_to_data_sharing.htm).

## Abbreviations

ADR: Adverse drug reaction
DLco: Carbon monoxide diffusing capacity
FVC: Forced vital capacity
IPF: Idiopathic pulmonary fibrosis
MDT: Multidisciplinary team
SD: Standard deviation
SE: Standard error
