# Supplementary material for: Longitudinal clinical outcomes in a real-world population of patients with idiopathic pulmonary fibrosis: the PROOF registry
Source: Respir Res. 2019 Oct 24;20:231. doi: 10.1186/s12931-019-1182-z (PMC6813978; doi:10.1186/s12931-019-1182-z)
Supplement: Supplementary file 1 — Additional file 1: Table S1. Percentage of patients with categorical relative decline in percent predicted FVC and DLco over 24 months compared with Month 0 (pirfenidone-treated population); Figure S1. Mean percent predicted FVC over time in patients that survived and in patients that had died at Month 24 (pirfenidone-treated population). [file 12931_2019_1182_MOESM1_ESM.pdf]

Additional file 1

Supplementary Table 1 Percentage of patients with categorical relative decline in percent predicted FVC and DLco over 24 months compared with Month 0 (pirfenidone-treated population)

	Month 3	Month 6	Month 12	Month 24
Relative FVC decline ≥10%, *n*/*N* (%)	20/144 (13.9)	26/145 (17.9)	31/131 (23.7)	20/58 (34.5)
Relative DLco decline ≥15%, *n*/*N* (%)	26/137 (19.0)	37/137 (27.0)	29/121 (24.0)	27/56 (48.2)

DLco carbon monoxide diffusing capacity, *FVC* forced vital capacity

Supplementary Fig. 1 Mean percent predicted FVC over time in patients that survived and in patients that had died at Month 24 (pirfenidone-treated population)

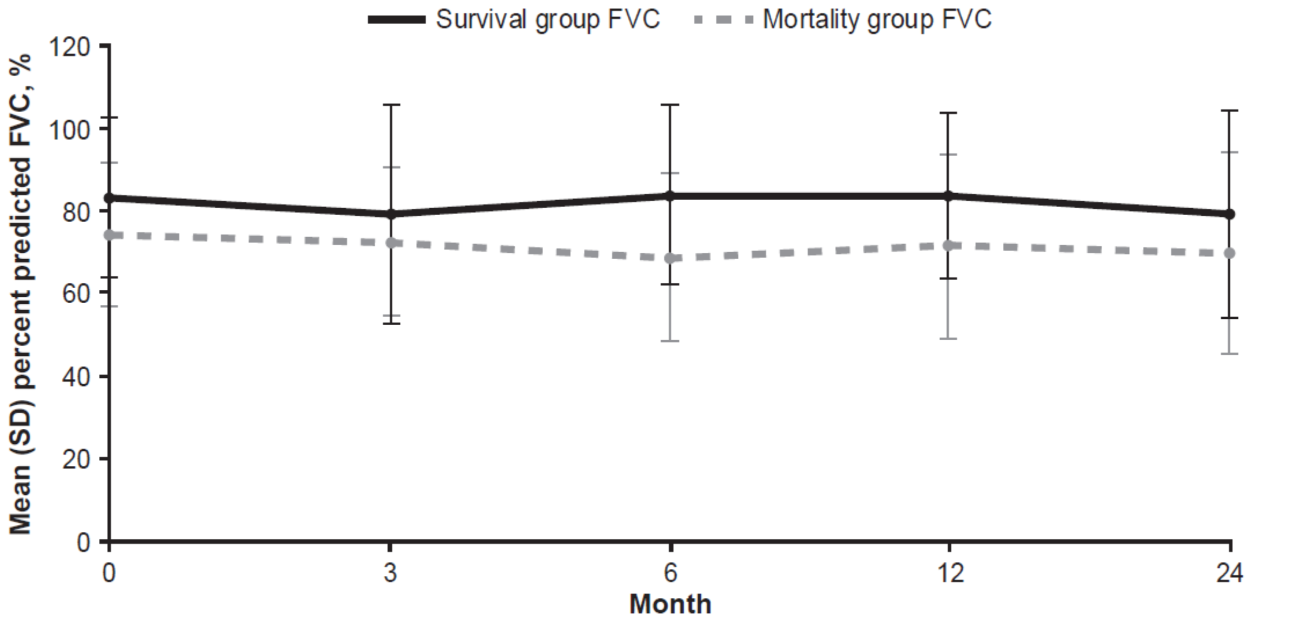

	0	3	6	12	24
Mean percent predicted FVC in patients alive at Month 24 (Survival group)	83.18 (*n* = 158)	79.04 (*n* = 134)	83.72 (*n* = 133)	83.65 (*n* = 128)	79.26 (*n* = 61)
Mean percent predicted FVC in patients who died at ≤24 months of follow-up (Mortality group)	74.36 (*n* = 47)	72.34 (*n* = 32)	68.53 (*n* = 30)	71.33 (*n* = 18)	69.71 (*n* = 7)

FVC forced vital capacity, *SD* standard deviation
